# Aging and Mentorship in the Margins: Multigenerational Knowledge Transfer Among LGBTQ+ Chosen Families

**DOI:** 10.1093/geronb/gbaf027

**Published:** 2025-02-15

**Authors:** Angela K Perone, Lindsay Toman, Beth Glover Reed, Tré Coldon, Ashlee Osborne, Justice Cook

**Affiliations:** School of Social Welfare, University of California, Berkeley, Berkeley, California, USA; LGBTQ Studies, Colgate University, Hamilton, New York, USA; School of Social Work, University of Michigan, Ann Arbor, Michigan, USA; School of Social Welfare, University of California, Berkeley, Berkeley, California, USA; School of Social Welfare, University of California, Berkeley, Berkeley, California, USA; School of Social Work, University of Michigan, Ann Arbor, Michigan, USA; (Social Sciences Section)

**Keywords:** Capital, Intergenerational, Power, Support

## Abstract

**Objectives:**

For LGBTQ+ communities, learning often happens among chosen families, including older adults. Building on Pierre Bourdieu’s theoretical concepts of capital (e.g., economic, social, cultural, symbolic) and queer theory of sexual capital, this article examines how LGBTQ+ chosen families share expertise to build knowledge and power across the life course.

**Methods:**

Using a transformative sequential mixed-methods design from a larger project, this subproject includes data from 6 intracategorical focus groups with multigenerational and multiracial LGBTQ+ participants (*n* = 37), including older adults, in a Midwestern community to center their voices, understand their experiences within and outside LGBTQ+ communities, foreground experiences of LGBTQ+ aging, and explore challenges and supports.

**Results:**

We identified 3 ways in which LGBTQ+ chosen families shared knowledge about various forms of capital: latent mentorship, bi- or multi-directional mentorship, and transgressive mentorship. We call these 3 types of knowledge sharing “mentorship in the margins,” in which knowledge is shared within and among communities whose intersecting positionalities both limit and expand ways to imagine mentorship for navigating structural barriers and social, economic, and political inequities, especially regarding shared housing, family formation, and marriage equality.

**Discussion:**

The breadth and depth of multigenerational transfers of knowledge across the life course demonstrate the centrality of multigenerational chosen families for LGBTQ+ communities as they age, especially among multiply-minoritized communities (e.g., transgender women, Black, Indigenous, and People of Color [BIPOC] same-gender-loving communities). Knowledge shared among chosen families also reflects how “mentorship in the margins” builds individual and collective power that helps LGBTQ+ communities survive and thrive as they age.

Many LGBTQ+ older adults came of age when sharing one’s sexual orientation or gender identity could result in state-sanctioned violence and loss of employment, children, and housing. Covert and overt discrimination led to what some scholars refer to as minority stress (Flatt et al., 2022; Meyer, 2003) that is further compounded by historical and structural inequities. LGBTQ+ older adults have also experienced historical pathologization related to their sexual orientation or gender identity. In response to rampant discrimination and threats of violence, many LGBTQ+ older adults formed networks of support through multigenerational chosen families (described more below) that provided literal lifelines as well as information about how to navigate the world as an LGBTQ+ person (e.g., Tester & Wright, 2017). While decades of community advocacy have led to significant advances for LGBTQ+ rights, LGBTQ+ communities continue to face discrimination in nearly all spheres of life—some to greater degrees than others given various intersections of their sexual orientation and gender identity with race, ethnicity, ability, and age, among other aspects of who they are. This study examines how multigenerational LGBTQ+ communities (and chosen families) share expertise and informal mentorship to build knowledge and power across the life course.

## Chosen Families

A term first coined by Weston (1991), “chosen family” refers to family-like networks that LGBTQ+ people form in addition to or in place of a legal and biological family (or family of origin). These networks can also include one’s community. As chosen families look different from person to person, some possess “broader community-type groups” (Jenicek & MacIntosh, 2022). In a study analyzing how LGBTQ+ people conceptualized family, researchers found that respondents expanded the definition of chosen family to include community and support groups (Hull & Ortyl, 2018). Chosen families are often created in response to unsupportive family relationships or in reaction to traumatic experiences that LGBTQ+ communities face based on sexual or gender identity—serving as both survival mechanisms and resistance to heteronormative family life (Dewaele et al., 2011; Hailey et al., 2020). Chosen family includes persons who bring closeness and strong ties (e.g., friends, classmates, colleagues, neighbors) (Gazso & McDaniel, 2015; Roberts et al., 2023) and intentional familial bonds created when same-sex couples marry or have children (Hull & Ortyl, 2018). Chosen families are deliberately constructed among people who provide support, trust, and reciprocity and may hold more power than a person’s blood and legal ties (Gazso & McDaniel, 2015; Hull & Ortyl, 2018; Roberts et al., 2023) but can also include looser but meaningful connections among community and support networks (Hull & Ortyl, 2018). For purposes of this paper, we define power as both a matrix of domination of multiple, interlocking systems of oppression and source of resistance (Hill Collins, 2008). While some chosen families may differ in including those with a “closer” relation to the individual, they are all similar in that they perform functions normally associated with families of origin but which may be inaccessible.

Chosen families can and often do provide formal and informal mentorship in identity formation, relationships, health, and life transitions (Kaufman et al., 2024; Levin et al., 2020), including widowhood (Valenti et al., 2023), and can help buffer adverse effects of stigma and homophobia (Gazso & McDaniel, 2015; Hull & Ortyl, 2018). This is especially true for communities of color. Chosen families for Black LGBTQ+ youth can provide mentorship that helps mitigate psychological distress by helping navigate systematic and race-based oppressions (e.g., teaching survival skills to withstand bullying) and building community to counteract social isolation (Hailey et al., 2020; Levin et al., 2020). Chosen families among LGBTQ+ older adults can provide informal mentorship that fosters connection and caregiving support in the face of health concerns, partner losses, and other developments across the life course (Hull & Ortyl, 2018). Chosen families can fill knowledge gaps that biological or families of origin may have otherwise provided about economic, social, or cultural power.

Moreover, chosen families often include multigenerational LGBTQ+ communities (e.g., Huynh, 2023; Sirdenis et al., 2019) that could span six generations, as visually depicted through what we call the Rainbow Generations Chart (see Table 1). The Rainbow Generations Chart includes three generations of LGBTQ+ older adults already identified by previous scholars (e.g., Fredriksen-Goldsen et al. 2024), including the Invisible, Silenced, and Pride Generations. We also identify three newly named LGBTQ+ generations (Caregiver, Equality, and Ze Generations) that capture unique experiences among LGBTQ+ historical cohorts that correspond with more mainstream generational categories. The Caregiver Generation represents a younger generation of emerging older adults whose historical cohort was heavily impacted by the AIDS epidemic of the 1980s and 1990s. The Equality Generation represents a generation of adults that aligns with Millennials who came of age during a period of various gains in LGBTQ+ equality (e.g., marriage equality, repeal of Don’t Ask, Don’t Tell and Defense of Marriage Act, increasing number of public figures coming out). The Ze Generation represents a cohort of younger adults that is coming of age during a time of increased gender fluidity reflected in public spaces and significant backlash toward gender diversity, transgender rights, and gender expression.

**Table 1.** Rainbow Generations Chart

LGBTQ+ generation name	Birth year range	Age range (as of January 2024)	Cohort experiences	Mainstream generation name
Invisible Generation	1934 and earlier	90 years+	Sexual and gender identities were “invisible”; WWII, Great Depression; increasing gender fluidity as women entered labor force during WWII; medical and legal institutions defined homosexuality as an “inclination”	Greatest Generation (born 1901–1924)
Silenced Generation	1935–1949	75–89 years	Lavender Scare; medical and legal institutions defined homosexuality as a “perversion”; Kinsey scale introduced	Silent Generation (born 1925–1945)
Pride Generation	1950–1964	60–74 years	Civil rights era; reproductive rights; rise of collective activism; Stonewall; creation of rainbow flag	Baby Boomer Generation (born 1946–1964)
Caregiver Generation	1965–1979	45–59 years	AIDS epidemic; DOMA; Don’t Ask, Don’t Tell	Generation X
Equality Generation	1980–1996	26–44 years	Marriage equality; repeal of Don’t Ask, Don’t Tell and DOMA; more public figures coming out	Millennials
Ze Generation	1997–2013	11–25 years	Gender diversity; fluidity; rise of discriminatory laws based on gender expression and identity (trans exclusions)	Generation Z

*Note*: DOMA = Defense of Marriage Act.

The Rainbow Generations Chart underscores how mentorship across multiple generations can occur (hence this article’s focus on “multigenerational” vs. “intergenerational”). While there has been an extensive amount of literature focusing on the role of mentorship within various social institutions, such as academic libraries (Mazari et al., 2021), formal mentoring programs within nonprofits (Burningham & Weiler, 2021), undergraduate student organizations (Hurley, 2016), and within families (Soler, 2016), research regarding the nuances of multigenerational mentorship within the LGBTQ+ community is scant. Although Burningham and Weller (2021) explore the health-related impacts of natural, meaning the relationships organically developed between friends and/or family members, versus formalized mentoring on LGBTQ+ youth, our study expands on LGBTQ+ multigenerational mentorship categorization by focusing on less formal mentorship. It also expands the concept of LGBTQ+ mentorship to include mentorship among multiple generations, including among different generations of older adults (as well as between younger and older generations). While mentorship among multigenerational LGBTQ+ communities can be both formal and informal, multigenerational LGBTQ+ chosen families may be especially well-equipped to provide knowledge transfer through informal mentorship.

This idea of a chosen family, including the broader community and across multiple generations, is also not exclusive to LGBTQ+ populations. For example, there is a longstanding tradition among Black communities to extend chosen family networks to neighbors or informal community support networks to cope with the difficulties that affect this demographic (Kane, 2000). Some argue that an entire community could be members of one’s chosen family (e.g., Stewart, 2007). In immigrant communities, some choose to incorporate community members as a part of their chosen families, especially for childrearing, to ensure spiritual development, cultural continuity, and financial and social support (Ebaugh & Curry, 2000). Disparities in power among minoritized communities based on race, ethnicity, sexual orientation, and gender identity have necessitated creation of chosen families—and perhaps even more so among LGBTQ+ communities with intersecting positionalities experiencing marginalization (e.g., racism, sexism, homophobia).

## Types of Capital

Multigenerational mentorship serves as a source of knowledge transfer about navigating various types of resources and power, which lends itself well to a study of multigenerational chosen families and capital. Capital explicitly foregrounds relationships of power and allows for an examination of the complex and nuanced ways in which LGBTQ+ chosen families share knowledge about these resources across generations. This article builds on a growing gerontological literature on how capital serves as a resource for older adults and aging in the context of social engagement (Boerio et al., 2023), retirement villages (Schwitter, 2022), volunteering (McNamara & Gonzales, 2011), health (Lu et al., 2023; Sun et al., 2017), and alcohol risks (Adnum et al., 2024).

Bourdieu describes four types of capital: economic, social, cultural, and symbolic. Economic capital is associated with money and wealth (Bourdieu, 1986) and comprises material wealth, property, and financial assets (Samanta et al., 2015; Xu & Jiang, 2020). Social capital refers to a social position or the advantages associated with networks of relationships and social connections (Bourdieu, 1986) that produce a durable, trustworthy, reciprocal, and resource-rich network of connections (Hussen et al., 2018, 2022) and includes institutionalized relationships such as families (Power, 1999), close friends, neighbors, or coworkers (Hussen et al., 2018), online networks (Venunath et al., 2023), civic engagement (Putnam, 2001), and informal channels or social credentials (Hussen et al., 2018). Li et al. (2023) describe how trans older adults address social marginalization and stigmatization by generating what they call authenticated social capital created by renegotiating social constraints and building alternative social networks and supports that assist them.

Cultural capital is thought to exist in three forms: (1) the embodied state, or long-lasting dispositions of the mind and body acquired across the life course; (2) via cultural goods (e.g., pictures, books), and (3) in the institutionalized state (e.g., educational qualifications) (Bourdieu, 1986). Yosso (2005) notes that cultural capital among communities of color and LGBTQ+ people can be aspirational (e.g., marriage equality), linguistic, navigational (e.g., maneuverability in institutions), and resistant (e.g., active resistance or more passively resisting by living one’s true and authentic life) in ways that resist heteronormativity and racism. Pennell (2016) adds that cultural capital can be transgressive and move beyond a boundary to deconstruct oppressive structures (e.g., chosen families, undocuqueer movement) and can include playfulness (e.g., drag culture, balls).

Building on Bourdieu, Green (2008, 2022) conceptualized sexual capital as the quality and quantity of attributes that produce erotic responses from others and can include embodied physical features (e.g., age, height, race, body size) (Montemurro & Hughes, 2024) and other aspects that can produce sexual desires associated with power. Symbolic capital emerges when other types of capital become legitimized (Bourdieu, 1989, 2000) by, for example, achieving hegemonic masculinity through sports (Tam & Kwan, 2024), marriage equality for same-sex couples (Winter, 2020), or legitimized symbols of equality like the pride flag (e.g., Gilmore, 2023). Symbolic power includes the ability to define and impose meaning and identify what is legitimate, whereas symbolic violence occurs when problematic meanings become an accepted norm (e.g., hegemonic masculinity, defining who is loveable) (e.g., Atkinson, 2024). Rutagumirwa & Bailey (2025) illustrate how body capital among older Black men (e.g., strength, functionality, age) shaped their sense of masculinity and whether their embodiment of masculinity was legitimized.

By building on these five concepts of capital, we can examine the following research question: How do LGBTQ+ chosen families share expertise to build knowledge and power across the life course?

## Method

This study incorporates six intracategorical (McCall, 2005) focus groups (everyone identifies within the LGBTQ+ category) designed to explore multigenerational and other differences among diverse LGBTQ+ populations. Participants from these focus groups (*n* = 37) were part of a larger study examining how to operationalize critical intersectionality frameworks (e.g., Crenshaw, 1989; Glover Reed et al., 2021; Hill Collins, 2008; Hill Collins & Bilge, 2020) in research in different contexts with different populations (e.g., LGBTQ+ older adults, Middle Eastern and North African [MENA] youth and adults, disabled adults, Black mothers, etc.). The impetus for this event and these focus groups arose from earlier focus groups with older LGBTQ+ adults (and especially two with Black same-gender-loving older men) and discussions in several support groups and advocacy organizations. Members expressed some envy and gratitude that younger members of the LGBTQ+ community were *not* experiencing similar types of discrimination and a history of needing to hide their sexual orientation or gender identity, but also some concern that they might not be anticipating challenges. They were interested in learning from younger generations, but also wanting to be helpful, and provide some of the kinds of supports that either they had in their younger years, or wished they had.

Focus groups are particularly beneficial for exploring complex phenomena and needs among minoritized communities (Redden et al., 2023) and when participants have something in common and share a stake in the issue being discussed (Barbour & Morgan, 2017). Focus groups allow participants to bounce off ideas, diversify opinions, and flesh out issues more comprehensively (Prasad et al., 2022). The larger project aimed to understand how people make sense of and respond to the positionalities that shape their lived experiences. Positionalities reflect one’s positions of power in relation to others in various social, political, and economic structures, cultural contexts, and interpersonal dynamics (e.g., Bilgen et al., 2021; Fresnoza-Flot & Cheung, 2024; Jackson et al., 2024; Martínez, 2023). Data from two prior intracategorical LGBTQ+ focus groups with Black same-gender loving older men (from this larger project) underscored the importance of multigenerational mentorship—and in different ways from mentorship outside the LGBTQ+ community. The research team thus subsequently conducted six multigenerational LGBTQ+ focus groups to further investigate multigenerational mentorship, among other topics (see Supplementary Material).

### Participants and Design

The intracategorical design focuses on the complexity of experience *within* a particular social position or intersection (McCall, 2005; Tinner et al., 2023) to explore complexity among LGBTQ+ participants who were diverse in age, race, gender, and sexual orientation—in particular how people make sense of and respond to the positionalities that shape their lived experiences. After some introductions, we asked explicitly about role models/or lack thereof, what influences positive or problematic efforts to support/care, larger influences on these interactions and how they were navigated, and then ideas for moving forward. See Supplementary Material for more details about the focus group protocol. While five of the six focus groups included five to six participants each, one group was larger in composition (*n* = 9) because it incorporated two participants who arrived 10 min late after focus groups had already been configured. It also included one participant who wanted to switch groups to be with his husband. In keeping with community-engaged research principles (that foreground shared power and equity in decision making [Ubri et al., 2024]), these additional participants were added to the same focus group to avoid disruptions and fracturing rapport with the other groups. Each group had representation from two or more generations. Group 1 consisted of participants from Silenced, Pride, and Equality Generations. Group 2 had participants from Pride and Equality Generations. Group 3 had participants from Silenced, Pride, Caregiver, and Equality Generations. Group 4 had participants from Caregiver and Equality Generations. Group 5 had participants from Pride and Equality Generations. Group 6 had participants from Pride, Caregiver, and Equality Generations. See Table 2 for participant descriptions. Each group also included one facilitator. Some focus groups included a separate notetaker or co-facilitator, which comprised a trained student further building methodological skills through this experience. The focus groups took place in two rooms provided by a community partner: five focus groups occurred in one large multipurpose room, and a sixth focus group occurred in a small meeting room.

**Table 2.** Narrative Participant Description

Participants	Description
Total participants (*n* = 37)	The total included a multiracial (Black, Latine, Native American, and White), multigenerational (age range 24–89), multigender (woman, man, genderqueer) group with diverse sexual orientations (same-gender-loving, gay, lesbian, bisexual, queer). Nearly half of the group comprised people of color (predominantly Black and to a smaller degree multiracial with Native American). More participants described themselves as male, than female (or woman), but some participants selected multiple gender categories and/or identified as genderqueer. While the overall group comprised participants from all Rainbow Generations (except Invisible Generation), it had fewer participants from the Ze and Equality Generations and more from the Caregiver, Pride, and Silenced Generations.
Individual focus groups	Individual focus groups reflect the composition described above for the overall participants. Nearly half of participants in each focus group included participants of color. Each focus group included at least two participants who described themselves as female or woman. Each focus group also included participants from at least three different sexual orientation positionalities. Each focus group included participants in the Equality, Caregiver, Pride, and Silenced Generation.

*Notes*: The table reflects a narrative participant description to better capture the complexity of participants’ intersecting positionalities, as self-reported from participants through multiple “check all that apply” and write-in categories from a demographic survey. While unconventional, we believe it aligns with critical intersectionality scholarship (e.g., Crenshaw, 1989; Glover Reed et al., 2021; Hill Collins, 2008; Hill Collins & Bilge, 2020) by presenting the complexity of intersecting positionalities as participants described themselves, which is sometimes harder to capture in separate table cells in a traditional table. It also aligns with larger strategies of knowledge sharing from this article about resistant and transgressive mentorship that resists dominant paradigms of knowledge transfer and presents an alternative example for sharing knowledge—in this case—a narrative participant description for other researchers to use when describing participant data.

Participants were recruited through purposive and convenience sampling through the main community partners who serve LGBTQ+ communities in the geographic region where the focus groups occurred. Community partner organizations served LGBTQ+ older adults, Black LGBTQ+/SGL individuals, and LGBTQ+ youth. Recruitment occurred through word-of-mouth, emails, flyers, and through an LGBTQ+-specific newspaper. Focus groups lasted approximately 2 hr. All focus groups were audio-recorded (with participant informed consent), de-identified, and transcribed by student research assistants. Participants received a $30 gift card for their participation. This project received IRB approval by a university’s institutional review board.

### Data Analysis

The research team incorporated a hybrid approach to thematic coding (Perone, 2023) in Dedoose (2021) that used inductive and deductive approaches (Houben et al., 2021; Portacolone et al., 2019). First, the team conducted open-coding to develop a thematic codebook that we used to carry out deductive coding. The codebook had 14 codes (i.e., barriers, caregiving, coming out, community, envisioning the future, faith/religion, intersectionality, marriage, mental health, mentorship, politics, relationships, and supports). While we organized the six focus groups around multigenerational LGBTQ+ knowledge transfer, they were also part of a larger study that had broader goals of understanding how people make sense of and respond to the positionalities that shape their lived experiences. Additional team discussions led to a subsequent focus on multigenerational mentorship and a second round of thematic coding that included a theory-informed analysis (Braun et al., 2016)—informed by five types of capital (economic, social, cultural, sexual, and symbolic). Capital provides a helpful theoretical framework for understanding how mentorship among participants was shaped by various types and relationships of power and intersecting positionalities. Coders produced research memos that were discussed with each other and the larger research team.

The research team engaged in reflexivity about their positionalities throughout the research project, including the larger study and prior focus groups that helped frame these six focus groups. Reflexivity involves “the recognition of the researcher’s particular social positioning and its impact on research” (Avishai et al., 2012, 397), by meeting often to discuss the analysis process. These conversations and reflections were captured through research team discussions and memos as further discussed in the Supplementary Material.

## Findings

We found that mentorship in LGBTQ+ communities happens in three significant ways, through what we call latent mentorship, bi- or multi-directional mentorship, and transgressive mentorship. These strategies of knowledge sharing reflect informal mentorship that occurred through what we call collectively “mentorship in the margins,” in which knowledge is informally shared within and among communities whose intersecting positionalities both limit and expand ways to imagine mentorship for navigating structural barriers and social, economic, and political inequities. Such barriers and inequities may limit more traditional forms of mentorship and may form the seeds for resistance and innovation to expand the possibilities for mentorship among chosen families. The findings illustrate numerous examples of mentorship in the margins that shared knowledge about various forms of capital (Bourdieu, 1986; Green, 2022; Pennell, 2016) among LGBTQ+ chosen families. Because capital centers concepts of power, it presents a helpful theoretical tool to understanding informal mentorship among multigenerational LGBTQ+ chosen families and how multigenerational LGBTQ+ chosen families share expertise to build knowledge and power across the life course.

### Latent Mentorship

Latent mentorship includes knowledge shared implicitly or informally (e.g., via actions, routines, etc.). Latent mentorship sometimes occurred around building knowledge and support about financial power, including through shared housing to avoid being unhoused. For example, an older transgender lesbian from the Caregiver Generation talked extensively about a person she lovingly called Grandma Annie who shared her home with people who needed temporary housing.

My friend Annie who died a number of years ago now... I learned a lot from her about family and community and having an open-door policy. I knew people who went to her place when they were in transition. And my house has become that sort of place. I have an attic. I had a number of friends over the years who are breaking up, moving back to town, you know–they finished school, figure they can live in my attic, find themselves, and leave. I learned the importance of open-door and community.

Not only did Grandma Annie provide a safe space for people who needed housing and extra support, her actions implicitly provided mentorship about shared housing. This participant replicated her experience with Grandma Annie by subsequently providing shared housing through available space in her attic. Many LGBTQ+ youth face homelessness, with 40% being forced out of their homes after coming out to their families (DeChants et al., 2021). According to the Trevor Project’s National Survey on LGBTQ+ Youth Mental Health (2021) over 28% of LGBTQ+ youth have experienced homelessness or housing instability at some point in their lives. This number increased when considering Native/Indigenous LGBTQ+ youth (44%) and transgender youth at roughly 39% (DeChants et al., 2021). Additional research has confirmed rising rates of homelessness among LGBTQ+ adults, including older adults (Wilson et al., 2020).

An older Black participant from the Pride Generation also referenced the informal exchange of mentorship regarding economic capital in response to younger LGBTQ+ individuals being evicted from their homes based on their sexual orientation and/or gender identity.

People get old, and they got extra spare bedrooms they might be wanting to rent out, and young people their parents kicked them out …. I would much prefer a lesbian, or even gay men…. the old people can supplement their social security and the young people won’t be hassled by who they have in their bedroom at night.

As with Grandma Annie, models of multigenerational shared housing among LGBTQ+ communities illuminate ways to build knowledge and power through economic capital that may not be centered around explicit conversations. Mentorship occurs by example and through actions of multigenerational shared housing models among LGBTQ+ communities that can help older adults with limited incomes make ends meet and provide safe and affirming housing for younger LGBTQ+ adults who have experienced eviction or other forms of housing discrimination based on their sexual orientation and/or gender identity.

Participants also discussed legalized marriage for same-sex couples as a way of creating financial security, therefore building economic capital. However, generational differences surfaced as LGBTQ+ older adults detailed the importance of marriage and questioned why younger generations are seemingly not prioritizing marriage as a financial resource. One older White participant from the Pride Generation stated, “I don’t want to say this as fact, when that might be a generational thing; I don’t hear a lot of younger queer people—or my peers aren’t talking about getting married nearly as much….they’re not buying into the institution in and of itself.” A younger Native American queer participant from the Ze Generation reiterated the value that a legally recognized marriage can create, pointing out the lack of enthusiasm for marriage among their peers:

I also just want to clarify. For me personally, marriage is really important. But it’s just like a generational thing amongst my peers–it is not as valued. When we were talking about creating networks and like what provides like security, I think why people are so anxious about marriage is I don’t think it provides the security that people think... that it may have been thought of to provide in the past.... I think people are thinking oh, I want to be able to secure my job, I want to be able to secure where I live.

Participants also reflected on the importance of social relationships within the LGBTQ+ community for fostering support with identity formation and knowledge about where to seek support and build social networks among LGBTQ+ communities. An older White gay man from the Silenced Generation discussed learning how to feel comfortable with his identity from an LGBTQ+ friend.

I don’t really have a role model. I have a friend whose been dead for years. He was someone whose scene I was nervous to talk to, he allowed me to talk. He taught me to be free, showed me how to accept who I was, re-showed me how to live. He’s like a role model to me. We were very close. He was like a father figure to me, not a father to me, but like a brother.

The examples above illustrate the complexity of mentorship about economic and social capital that occurs among LGBTQ+ communities across ages. They described learning strategies for navigating economic insecurity and LGBTQ+ community by watching other LGBTQ+ people and learning by example (e.g., shared housing) as well as informal conversations about ways to leverage economic security and social connections within or outside the legal institution of marriage.

### Bi- or Multi-Directional Mentorship

Bi- or multi-directional mentorship includes knowledge shared across two or more people in less conventional or expected directions (e.g., younger LGBTQ+ adults mentoring newly out LGBTQ+ adults about social networks). For example, several participants described how they learned about where to find new social networks, including dating, from LGBTQ+ individuals that were both within their age cohort and those younger (or older) than them. For example, a White bisexual participant from the Equality Generation illustrated mentorship around social and sexual capital when she described how a same-aged friend who “seemed so much more experienced and knowledgeable” showed her how to find the LGBTQ+ community through Craigslist where “a whole group of folks were using it to list events where [one] can go and meet people who are interested meeting someone to date or have friends, or, whatever in terms of LGBT spaces.” An older White participant from the Silenced Generation also described mentorship about LGBTQ+ social networks from a younger gay man from the Pride Generation as he was coming out as a gay man later in life.

I had an interesting, uh, I guess intergenerational mentoring. One of my students at the time, graduated in the mid ’70s, and I didn’t know he was gay then. I was closeted .… When I first came out, I was coming out like, ’89, ’90 or something, we got together as he was visiting in town—visiting his parents. We went out to dinner somewhere and came out to each other—conversation just kind of went in that direction. Here was this 33- or 34-year-old mentoring a 49- or 50-year-old, ’cause he was telling me about things like what to look for in relationships and cluing me in about the club scene.

LGBTQ+ chosen family provided opportunities for sharing knowledge about building LGBTQ+ social communities and networks (i.e., social capital), which were especially important as people (across the age spectrum) came out. These social relationships allowed individuals to learn from one another about how to be present within dating networks and how to live confidently as an LGBTQ+ individual.

Participants also discussed how they shared knowledge and power within LGBTQ+ networks about cultural capital related to religious institutions. For example, another White lesbian from the Caregiver Generation shared that she became “a born-again Christian” in college and subsequently led Bible studies while married in her late 20s. When a younger woman in her church sought guidance because she was having thoughts and feelings for women, this participant found herself at a “point of conflict” and told the younger woman that “I know that this is what this church teaches, that this is a sin, but I don’t necessarily think that that’s true [and that] God is going to love you no matter what.” This participant later moved from Texas to California where she came out as a lesbian later in life. She later reconnected with the younger woman from her Bible study and shared her own coming out story and that she was now happily married to a woman. This participant’s experience illustrates how two lesbians questioning their sexuality in restrictive churches navigated these cultural norms in an institutionalized state (i.e., the church) and how these conversations left long-lasting dispositions of the mind and body across the life course. These emerging lesbians engaged in bidirectional (and latent) mentorship as they both navigated their sexuality inside and outside the church.

Bi- and multi-directional mentorship also occurred around relationship norms and sexual agency that transferred knowledge and power about sexual capital. For example, one participant described mentorship from a younger gay man who had come out before him about the “clubbing scene.” He added that he later “mentor[ed] … a young man” and “advised him to go at [his] own speed and [not] let anyone rush you into doing things you’re not ready for, whether it’s sex, or relationships.” The younger man later told him that he was the first person who “took him under his wing” and “gave him perspective.” Here, bidirectional mentorship occurred when younger LGBTQ+ folks mentored older LGBTQ+ friends who were navigating a new sexual market as newly out and as LGBTQ+ participants shared knowledge and support about healthy relationships. Another participant described mentorship from a gay friend about leaving an abusive relationship:

I had a person that came into my life at a difficult moment. A relationship that I was involved in that was extremely abusive and I was trying to figure my way where I needed to get away, escape, and as a result this individual gave me some sound advice based upon their own personal experience, their own personal journey …. he said, “One day you’ll meet somebody, and that person will bring so much good and so much positive energy into your life that you’ll forget that that person ever existed,” and of course I couldn’t agree with that at that particular time, but it did eventually occur in my life. As a result of his mentorship, then I had somebody who were in the same kind of situation that I was that I got an opportunity to share my experiences with and as a result of sharing my experiences, it allowed them not to sink into the depression and the frustration that I actually experienced that caused me to have to reach out for something artificial in order to help me make it through the moment and as a result they didn’t have to do that.

The participant here relied on another gay man who escaped an abusive relationship to provide him perspective and hope for something better. This participant was then able to mentor another gay man in a similar situation and helped him find alternative resources that supported positive mental health and well-being. Research suggests that as many as one in 4 gay men, and 4 in 10 bisexual men have experienced intimate partner violence in their lifetime and that they can experience unique features of violence related to being gay or bisexual, including sexual orientation disclosure, unprotected sexual intercourse, homophobia, internalized homophobia, and challenges finding culturally responsive support groups and services (Callan et al., 2021). Mentorship about intimate partner violence can build sexual capital by arming LGBTQ+ folks with knowledge of supports, resources, and beliefs that they are worthy of love and relationships free from violence.

### Transgressive Mentorship

Transgressive mentorship reflects knowledge and power shared that deconstructs oppressive structures (e.g., sharing knowledge about LGBTQ+ symbols of gay [or straight] pride or that defy traditional gender norms). While discussing family formation, one participant from the Pride Generation described how her partner and she decided to have a child in the 1980s when same-sex couples were less likely to raise children. Her daughter is married to a woman, and they have two children. The participant described how because she was “on the cutting edge of the lesbian baby boom,” she became a source of information for other lesbian couples seeking to have children, including her daughter from the Equality Generation. Another participant (an older Black gay man from the Caregiver Generation) described how seeing his biracial gay friends raise two adopted children changed his perception of family formation and gave him a sense of what was possible as it forced him to “think and your predisposition is all wrong.” He added that “they have a big cookout, and they have about 30 or 40 of us brothers from around the country come” and that this cookout shows how two gay men (one of them Black) can raise kids and that “it works beautifully … better than most families I’ve known that are heterosexual.” Seeing other same-sex couples raise children became another form of de facto mentorship about family formation. While the lesbian couple “on the cutting edge of the lesbian baby boom” describes more overt mentorship with other same-sex couples, the mere existence of their family in the 1980s also served as a cultural model and form of resistant and aspirational cultural capital that helped families like them accrue cultural capital as more same-sex couples had children. Similarly, the other participant described more subtle mentorship about family formation that occurred while attending a biracial couple’s cookouts with their children. Mentorship, thus, about family formation among same-sex couples occurred explicitly and implicitly and reflected ways in which the mere existence of same-sex couples transferred knowledge that created resistant and aspirational cultural capital.

Several participants also discussed LGBTQ+ visibility in the workplace as a form of symbolic capital, including an older White lesbian from the Pride Generation who worked in the automotive industry.

One of my role models was a woman [who] worked at Ford Motor company, and she was probably the most senior out LGBT person at Ford while she was working there ... And I really admired her. She worked long hours. She was a hell of a welder. And I got to introduce her once at one of our educate your co-workers about LGBT stuff so that was kind of a fun thing to do.

Here, she learned by watching an older lesbian trailblazer at work engage in employment that defied gender norms about women in the workplace. As this out lesbian worker was increasingly recognized as a valued employee, the participant witnessed a process of legitimization in a job where women often struggled for recognition that blended latent and transgressive mentorship.

## Discussion

### Mentorship in the Margins

LGBTQ+ chosen families represented a wide diversity of social networks, role models, and mentors who informally provided information about power through economic, social, cultural, sexual, and symbolic capital. In many instances, this informal mentorship infused multiple forms of capital at once. Mentorship occurred bidirectionally, within generations, and across generations. Findings from this project illustrate how mentorship within LGBTQ+ chosen families often occurred “in the margins” and in ways that defied traditional dyadic and didactic forms of mentorship, particularly in shared housing, family formation, and marriage equality. Mentorship in the margins presented avenues for building knowledge and power across the life course for multigenerational LGBTQ+ chosen families.

Shared housing served as an important source of mentorship in the margins that provided an economic and social safety net as well as social networks to share resources. Mentorship about shared housing was sometimes described as latent, as LGBTQ+ chosen families learned from each other through action and not necessarily a step-by-step verbal tutorial or other more formal discussion. Participants also described how shared housing benefited LGBTQ+ chosen families across generations and how they learned about building capital through shared housing in bidirectional or multidirectional mentorship. Shared housing provided economic security for LGBTQ+ older adults and housing security for younger adults while also building multigenerational social support. Spaces of shared housing, thus, became sources of multigenerational knowledge about economic and “authenticated” social capital (Li et al., 2023) that built alternative social networks for support. Shared housing among LGBTQ+ chosen families also reflects resistant (Yosso, 2005) and transgressive (Pennell, 2016) forms of cultural capital by passively resisting dominant family housing arrangements and arguably deconstructing oppressive structures that leave many LGBTQ+ youth and adults unhoused (e.g., DeChants et al., 2021; Wilson et al., 2020). Shared housing presented alternative paradigms for economic and housing support that deconstructs oppressive housing structures that rely on biological families to provide the economic and housing safety net for each other and thus served as an important source of understanding and building knowledge and power.

Family formation comprised another area of mentorship in the margins that blended latent, bi- or multi-directional, and transgressive mentorship. Latent mentorship occurred as same-sex parents simply lived their lives (e.g., hosted cookouts) and showed other LGBTQ+ chosen families new possibilities for family formation. These relatively innocuous acts also comprise transgressive mentorship by deconstructing oppressive structures that center heteronorms around family formation (Dewaele et al., 2011; Hailey et al., 2020). Several older lesbian and gay participants proudly shared how they served as cultural representations for same-sex parenting and provided concrete knowledge to other LGBTQ+ chosen families about the process of building families with children. They also discussed how their trailblazing efforts formed the seeds for new multigenerational social networks around family formation that presented bi- and multi-directional mentorship to share expertise and build knowledge and power across the life course. These examples seemed particularly salient among lesbian participants and gay men of color whose multiply-minoritized positionalities regarding sexual orientation and gender or race required creative and multifaceted mentorship strategies about family formation. Many of the older participants describing mentorship around family formation were building this capital at a time when LGBTQ+ folks faced heightened discrimination as same-sex parents. Several younger participants from the Equality Generation expressed gratitude for the knowledge shared and the trailblazing efforts of these earlier same-sex parents.

Mentorship in the margins also surfaced around marriage equality. Participants discussed how marriage equality presented different visions of cultural capital across generations, which helped share expertise and build knowledge and power across the life course. For older generations, marriage equality was a decades-long battle for equality that also embodied a sense of pride and achievement. Its legitimization reflected the symbolic capital that they had long desired. It also represented a form of economic capital that helped ameliorate decades of inequities stemming from benefits attached to marriage. While some younger participants acknowledged the benefits of marriage equality in building economic capital, they tended to express a different vision of marriage equality—one in which their future lives were not dependent on this cultural, institutionalized state (Bourdieu, 1986). They described aspirational cultural capital (Yosso, 2005) and transgressive cultural capital (Pennell, 2016) in which legal benefits are not tied to marriage but bestowed upon citizens regardless of marital status. Discussions about marriage equality revealed fissures in mentorship and knowledge sharing among LGBTQ+ chosen families. Different generational experiences shaped what knowledge folks wanted to transfer and how they wanted to share it. Participants from the Silenced and Pride Generations were more insistent on underscoring the economic and symbolic benefits of marriage equality for younger generations, whereas participants from the Equality Generation were interested in sharing paradigm-shifting ideas about marriage that decoupled legal benefits from marriage altogether. Mentorship in the margins still occurred but in complex ways regarding this issue. Older participants were proud to serve as cultural representations of marriage equality through latent mentorship whereas younger participants were invested in transgressive mentorship that shared knowledge about new ways that LGBTQ+ chosen families could think about marriage (and relationship-building overall).

The breadth and depth of multigenerational knowledge sharing across the life course demonstrate the centrality of chosen families for LGBTQ+ communities as they age, especially among multiply-minoritized communities (e.g., transgender women, Black, Indigenous, and People of Color [BIPOC] same-gender-loving communities) navigating intersecting systems of oppression. It also underscores potential benefits of expanding traditional notions of families of choice beyond close networks to include looser but meaningful community supports (e.g., Hull & Ortyl, 2018) and how expanding this definition itself reflects a form of transgressive resistance to what and who constitutes “family” among LGBTQ+ communities. For many, biological families are families based on blood because society has determined that blood is a substantial connection—even when individuals related by blood have no otherwise meaningful connections. For many LGBTQ+ participants, the infrequent but meaningful mentorship interactions among community members and role models redefines what constitutes meaningful or “close” and by extension the possibilities of a chosen family. Knowledge shared among such chosen families also reflects how “mentorship in the margins” builds individual and collective power that helps LGBTQ+ communities survive and thrive as they age. Mentorship in the margins is complex and multifaceted but provides a new way to consider how knowledge transfer occurs, particularly in communities whose intersecting positionalities leave open new possibilities for envisioning mentorship.

### Limitations and Future Directions

One limitation of this study centers on a lack of perspectives due to the reliance on community partners to disseminate information and recruit participants. While working with diverse community partners helped ensure heterogeneous focus groups, it also meant that our focus group participants likely over-included participants who were more connected—or at least connected in some way to the community partners helping with outreach. This shared background among study participants may have created a reluctance for individuals not affiliated with the community programs to participate. Another related limitation was that because several focus groups occurred in the same—albeit large—room, it was sometimes difficult to hear other speakers at times and some participants occasionally had to repeat themselves to be heard, which may have hindered rapport in some groups. Nevertheless, prioritizing focus groups as the main source of data collection provided the opportunity for the research team to learn about lived experiences directly from participants and gather data that built off other participants’ experiences. This produced findings (and new ways of conceptualizing mentorship) drawn from participants’ experiences that built on methodological strengths of focus group conversation (McCorkel & Myers, 2003). Another limitation of this paper is its limited attention to intersecting systems of oppression as it relates to multigenerational mentorship. Given the complexities and nuances in this data, we explore this issue more in another paper that uses intersectional theories about domains of power to further examine how intersecting systems of oppression shape challenges and supports from an intersectional lens.

Despite these limitations, this article provides an innovative paradigm to consider informal mentorship that may be more common among underrepresented and multiply-minoritized communities, including LGBTQ+ communities, who may have limited access to traditional forms of capital throughout the life course. Latent, bi- and multi-directional, and transgressive mentorship also present three new forms of mentorship that can be used in future studies focused on mentorship within marginalized populations. Altogether, these concepts can further gerontological scholarship by presenting new possibilities to study ways in which mentorship shapes aging—and how informal mentorship, in particular, may shape life experiences, individual development, and community supports across the life course. These findings also foreground new possibilities for LGBTQ+ multigenerational programming and activism that center informal mentorship. Analyzing mentorship within the margins can aid in identifying pathways and possibly developing programs for historically marginalized populations to receive alternative forms of guidance. Finally, this research introduces a novel Rainbow Generations Chart that builds on LGBTQ+ aging research that previously identified three generations of LGBTQ+ older adults (Invisible, Silenced, Pride) (Fredriksen-Goldsen et al. 2024) by incorporating an emerging generation of older adults that came of age during the AIDS epidemic (Caregiver Generation) as well as two newly named LGBTQ+ generations (Equality and Ze Generations). Additional gerontological research is needed to examine how informal mentorship shapes challenges and strengths and how these newly described LGBTQ+ generations can build multigenerational knowledge and supports for LGBTQ+ older adults and LGBTQ+ communities generally.

## Supplementary Material

gbaf027_suppl_Supplementary_Materials

## Data Availability

Due to IRB and ethical restrictions with the community partners and participants, we are currently unable to share the raw qualitative data. However, examples of the coding process and focus group protocol are available upon request. This study was not preregistered.
