# Supplementary material for: Aging and Mentorship in the Margins: Multigenerational Knowledge Transfer Among LGBTQ+ Chosen Families
Source: J Gerontol B Psychol Sci Soc Sci. 2025 Feb 15;80(6):gbaf027. doi: 10.1093/geronb/gbaf027 (PMC12067074; doi:10.1093/geronb/gbaf027)
Supplement: gbaf027_suppl_Supplementary_Materials [file gbaf027_suppl_supplementary_materials.docx]

***The Journals of Gerontology, Series B: Psychological Sciences and Social Sciences* Supplementary Material: Perone et al. Aging and Mentorship in the Margins: Multigenerational Knowledge Transfer among LGBTQ+ Chosen Families.**

While this paper reports data from six intracategorical, multigenerational LGBTQ+ focus groups, these focus groups were part of a larger multi-method project, using a transformative framework (Mertens, 2012) with a sequential multi-methods design (Creswell & Plano Clark, 2017) and social justice goals. A major purpose for the larger study (involving 31 focus groups with diverse memberships, some intra-categorical [with shared positionalities] and some inter-categorical [with diverse positionalities]), was to explore systematically how to operationalize critical intersectionality frameworks and methods in research in different contexts with different populations, with work in earlier phases informing later approaches. Themes identified in earlier LGBTQ+ older adult focus groups led to the focus in these groups: on mentoring and cross-generational communication.

The six intracategorical multigenerational LGBTQ+ focus groups for this sub-study arose from data from two prior intracategorical focus groups with Black same-gender-loving older men. In those prior two focus groups, participants expressed strong interest in more multigenerational conversations with LGBTQ+ communities and urged the researchers to conduct additional focus groups that included LGBTQ+ of different ages and races. Participants expressed some envy and gratitude that younger members of LGBTQ+ communities were not experiencing the same types or degree of discrimination and need to hide their sexual orientation and gender identity that they had in prior decades and some concern that they might not be anticipating future challenges or risks. They were interested in learning from younger generations while also providing supports and guidance. The lead researchers from the prior two focus groups included an older Black heterosexual man, a white queer woman in mid-adulthood, and an older white heterosexual woman.

Half of the facilitators identified as BIPOC, and two were over 60 years old. Facilitator notes include seating charts with information about how participants described themselves, key themes, and deviations from the focus group protocol. The two oldest facilitators had many years of group facilitation and focus group experience and were founding members of the Critical Intersectionality Learning Community. The other eight facilitators engaged in two training sessions to prepare for group facilitation. First, they participated in a focus group as members, experiencing the protocol to be used in the groups they were preparing to facilitate. They debriefed their experiences as participants and critiqued the protocol and its implementation. They read several articles about focus group facilitation, and in a second session as a group, they reviewed and practiced implementing the protocol, recording, and note-taking procedures.

Intracategorical focus groups took place in a community setting and were preceded by a meal together to build rapport. When people arrived, they completed a short sign-in that asked them to identify their race, age, and gender, which were used to create multiracial, multigenerational, and multigender focus groups. All focus groups had multigenerational, multiracial compositions. See Table 2.

The focus groups took place in two different rooms provided by the community partner: a larger multipurpose room and a smaller meeting room. Five of the focus groups occurred in the large multipurpose room, whereas a sixth focus group occurred in the smaller meeting room. While we would have preferred six different rooms for the focus groups, the community organization provided the two rooms they had available, which meant that five of the six focus groups occurred in the same (albeit larger) room. This created some challenges with sound for some of the five focus groups. However, facilitators and co-facilitators helped repeat questions and responses when necessary. Each focus group also had two recording devices, which helped capture the audio for each focus group, even with occasional background noise.

We learned in earlier work from the larger project that it is important to provide some core definitions and allow participants to explore individually some core concepts, positionalities, dimensions, and contexts in their lives that were expected to be relevant in the focus groups. For these groups, we used a shorter version of a questionnaire used in previous groups. Participants completed this questionnaire after all questions were answered about goals, confidentiality, and general norms and procedures.

The questionnaire asked about the language they used to describe their gender and sexuality and other positionalities (e.g., race/ethnicity, economic and dis/ability status and health), and some current social contexts (partner status, people living with, giving and receiving caregiving). These were followed by a series of scales exploring experiences of everyday discrimination (implicit and explicit biases), mental and physical health, and vigilance to prepare for discrimination. After each set of scales, participants were asked what they thought about the reasons for the items they had checked; these included many positionalities or other characteristics associated with positionalities or forms of discrimination.

## **Establishing Norms**

Focus groups require that norms be developed to support open and diverse participation (Roller & Lavrakas, 2015). Before starting the focus group questions, facilitators shared desired norms and procedures for good and shared communication (e.g., listen and try to understand before responding; share one’s own experiences, take turns while talking and encourage others; share what you feel comfortable sharing; let us know if you feel uncomfortable; let us know if there is something you said that needs be deleted from the audio recording). Participants added ideas and asked procedural questions during this time. Facilitators also shared goals for the groups (intergenerational dialogue, discuss different aspects of our lives and who we are, key aspects of health and well-being, and how we cope with life experiences). Informed consent and confidentiality procedures were explained during the meal. After the meal, participants signed informed consent documents and were given a document to keep with contact information if they wanted to follow up.

## **Focus Group Protocol**

We began the focus group discussion with a light-hearted icebreaker about choosing an alias, but also to allow participants to share why they chose it and what it means/meant to them. We anticipated that these would vary considerably across generations and contexts. We also pulled this icebreaker from a recommended focus group facilitator’s guide from a national LGBTQ+ organization.

Participants then introduced themselves in two ways: a) by imagining they were meeting someone they wanted to know, for the first time, so that they could learn important things about the participant; and b) by sharing what led them to participate in this event and what they hoped to get out of the experience. The group then discussed what they had noticed in terms of similarities and differences among them, about themselves and their goals for the session.

The next topics were about connections, tensions, and contexts. Facilitators noted relevant themes from earlier discussions (e.g., multi-generational examples, respect, safety, peer pressure, resilience or struggle and despair) and goals for sharing lives and relationships in different contexts. The first set of questions was about (a) role models/mentors or lack of them [an LGBTQ+ role model or mentor, benefits, challenges] or when the participant had served these roles for others; (b) examples about how they do, or do not, care and support each other and what influences these (what creates stressors and difficulties, or supports positive experiences).

Part three focused on larger influences for and consequences of all the above (e.g., factors other than LGBTQ+ selves that influenced situations, examples of how they positively or negatively navigated them, barriers encountered and how they addressed them).

Part four then focused on moving forward, with ideas about the kinds of communities and supports participants would like to have in the future, for themselves or others. This involved considering locations/living environments and sources of giving/receiving support.

And finally, group members identified what were important things talked about in the group, and what they would like to see happen next (follow-up).

## **Reflexivity**

The research team employed self-reflexivity throughout the research project. Reflexivity is a process of researchers’ looking inward, questioning one’s taken-for-granted assumptions, and potential biases or standpoint(s), and how our roles, experiences, and perspectives shape knowledge production (e.g., Flores-Flores et al., 2023; Lowers et al., 2024). These processes helped us interrogate our positionalities and thoughtfully engage with the data and research process. Like the intracategorical focus groups, the authors reflect a multigenerational, multiracial, and multigender research team whose intersecting positionalities were incorporated throughout the research process. We have incorporated individual positionality/reflexivity statements that describe our intersecting positionalities, lived experiences, and approach to research below.

### ***Author 1 Positionality Statement***

As a community-engaged and critical scholar, I am invested in elevating voices of multiply minoritized communities through various forms of research collaboration. I recognize the value of scholarly and community expertise based on diverse forms of evidence and knowledge. I have received four graduate degrees and eleven years of advanced graduate training that shapes my empirical and theoretical approaches to research. But I also have over forty years of lived experience as a white queer woman who has lived in both rural, urban, and suburban communities for extended periods of time. I practiced law for nearly ten years before going back to graduate school to pursue a Ph.D. in social work and sociology. I also served for five years as the founding executive director for a small nonprofit in Michigan—the only nonprofit in the state to focus on services and advocacy for LGBTQ+ older adults—and as a senior health policy fellow in the United States Senate and Centers for Medicare & Medicaid Services. This blend of lived experience with professional and academic training also shapes my approach to research—by spurring a passion for research that often involves community members throughout the process and that has timely real-world implications.

I am an interdisciplinary scholar primarily trained in law, sociology, social work, gerontology, and public health. This interdisciplinary background further informs my approach to research and dissemination. While I tend to gravitate toward macro-oriented research questions and solutions, my interdisciplinary training has also provided a multi-level background that permeates my work and facilitates multi-level reflections throughout data collection, analysis, and dissemination. I aim to connect multiple levels of research, when possible, to expand understandings of complex social, cultural, economic, and political phenomena that shape the lives of multiply minoritized communities across the life course, including LGBTQ+ older adults.

During this particular research project, I engaged in reflexivity during data collection, analysis, and dissemination as I considered my role as an LGBTQ+ community member and researcher that is shaped by the experiences described above. I drafted research memos and other narratives and also engaged in multiple conversations with various team members, across the project on how my (and other team members’) intersecting positionalities and lived experiences shape our approach to data collection, analysis and interpretation, and dissemination.

### ***Author 2 Positionality Statement***

As a feminist researcher, I believe that the people studied should tell their story in their own words. My lens depicts social science research as an interpretive process from which I gain perspectives from the communities I study through the relationships created between participant and researcher (Gorelick, 1991). The knowledge created by social scientists must be products inspired by their subjects to eliminate distorted claims made by privileged researchers (McCorkel and Myers, 2003). This frame of analysis also encourages researchers to pay close attention to a participant’s social location and to neglect a master narrative. For many years, social scientists were guilty of creating “master narratives,” which refer to “the cultural frameworks that limit and structure the way that stories are told in order to support the status quo and the dominant groups’ perspective on reality” (McHugh, 2014, 151). However, it is pivotal that researchers practice reflexivity throughout the study and consider how their own positionalities impact their ability to analyze the data.

As a member of the LGBTQ+ community, coding the data felt familiar. The concept of a chosen family and mentorship in the margins was implicitly understood. However, as a white woman from a middle-class background, there were aspects of the conversations centered on racial discrimination and economic hardship that I understood only theoretically. I have not experienced racial prejudice due to my white privilege, and I have never been kicked out of my home based on my identity. Reflecting on these differences was vital throughout the analysis process.

### ***Author 3 Positionality Statement***

My professional/PhD training in community and clinical psychology, and years of teaching social work-related topics helped to hone skill sets and conceptual frameworks relevant for this work, but I came to emphasizing theorizing and research methods focused on critical justice through many types of community-based work to address injustice and work for justice. I was involved in anti-poverty initiatives in the 60’s, diversifying curriculum and teaching methods in K-12 curriculum and teaching, and in the 70’s addressing housing issues and community-based substance abuse prevention. I increasingly recognized that much available knowledge centered the perspectives of those with the most privilege, producing biased knowledge, and I began to work on illuminating these biases and developing frameworks and methods to try and fill some gaps. Initially, I focused on gender and racial differences in leadership and group composition in relation to theories of group development, group effectiveness and social change.

As a child, I was mostly economically secure in a semi-rural environment, but with limited academic resources, and I was the first on both sides of my family to get a college degree. I am white with mostly northern European ancestors, but grew up in an environment that attracted immigrants, including Holocaust survivors, and native Italian and Spanish speakers, with a small but strong and long-time Black community. As a very non-traditional “girl” in the late 1940’s and through the 1950’s, plus having a grandmother who was a social justice warrior (especially focused on race/ethnicity, class and religion), I recognized multiple inequalities and their impacts from a very early age. These were all heightened while navigating college, graduate school, and various jobs and professional positions where I was often the only woman or one-of-a-very-few, with even fewer from BIPOC categories, and then with experiences within a multi-racial family-of-choice. A great deal of mentoring, with a few notable exceptions, when it happened at all, happened among peers because it was minimally available otherwise. I was also involved in helping to develop Women’s, Gender, and Cultural Studies, more explicit systems for mentoring and removing barriers, and what we now call DEI policies and procedures. And now I am in the oldest generation so am increasingly navigating increased social and physical challenges.

For 15 years, I was PI for the Critical Intersectionality Learning Community (CILC), working with diverse colleagues to develop theory, research methodologies, and strategies that attended to diverse interacting systems of power, to work for justice. We systematically explored similarities and differences in mixes of positionalities, research and analysis methods, in multiple critical justice arenas (e.g., domestic violence among SE Asian immigrant families, mental health issues among young African American men). We worked to implement group and decision-making processes that honored critical intersectionality principles among diverse community-members, students, and faculty. The CILC helped to sponsor and implement these focus groups, drawing on lessons learned from all the above.

### ***Author 4 Positionality Statement***

As a Black, Queer person, I have always sought to uplift the experiences and stories of the people who look and live like me. I have been aware that LGBTQIA+ individuals are constantly put in positions where their circumstances are rendered invisible and, therefore, unimportant. This awareness has structured many choices in my life including my experiences as a Programming Intern responsible for planning social activities for Queer students during my undergraduate education and the years I spent as a Legal Assistant for an LGBTQ advocacy nonprofit, as both positions allowed me to serve this population as best I could. This awareness has also pushed me towards my training to be a social worker who specializes in Black, Queer populations.

Considering mentorship, furthermore mentorship across generations, the role it plays in the survival of LGBTQIA+ populations becomes apparent. This is especially true for BIPOC LGBTQIA+ people, who, because of their identity, must rely on “chosen families” to understand the ways of the world. Through connection with “fictive kin,” people can give and receive emotional support and wisdom from those around them, serving as a protective factor against many harms. Acceptance from family and friends is still a struggle for many BIPOC LGBTQIA+ people and until a time of universal support is reached, chosen families will continue to be important for support and advice on how to traverse the world while Queer. Concerning this, it is crucial to understand and explore the variety of ways mentorship takes form in these populations. Through this, we can understand how intergenerational learning works for LGBTQIA+ people and shed light on the resilience these individuals show as they pool their resources and experiences to support one another. My identity as a Black, Queer person lends itself to this research as I have had to gain insight and knowledge from similarly identified communities to grasp what it meant to be Black and Queer and how to “move” accordingly. However, my experiences have also been marked by large amounts of familial and friend support, rendering my knowledge of all the roles chosen family can fulfill, in the realm of mentorship, lacking.

### ***Author 5 Positionality Statement***

As a recent black female graduate pursuing a career in medicine, I have encountered various overlapping themes that have shaped my current journey. I have personally experienced how my appearance has influenced how I’ve been treated professionally and socially. I am deeply committed to understanding and addressing the complex issues surrounding the marginalization of certain communities in today’s society by listening to and learning from the real-life experiences of those around me, regardless of their similarities or differences.

When considering mentorship in the context of equitable healthcare, it is crucial to recognize the significant impact healthcare can have on one’s life experiences, especially for same-gender-loving adults. As the discussion around gender-affirming care and healthcare rights continues in the US, it’s essential to hear the stories of those who have faced obstacles in healthcare and have advocated for themselves or others to receive fair and high-quality healthcare, regardless of their race, gender, or sexual orientation. I utilize the lens I have as a black woman living in my majority culture to guide my conceptualization and approach to this project. I am a firm believer that understanding the influence of capital is vital to synthesizing the experiences of those who have had to advocate for themselves louder, stronger, or longer in order to access the same level of care or services as their counterparts. My black identity directly shapes my approach and contributions to this project as we assess the impact of mentorship in the margins for LGBTQ+ adults through a plethora of focus groups and analyses using Bourdieu’s capital framework.

### ***Author 6 Positionality Statement***

The purpose of this statement is to establish a connection between my personal background, belief system, and how these factors shape my research interests and approaches.

Some experiences and identities that have shaped my worldview are that I am queer, white, a young adult, an advocate for the aging population and grew up in a low-income household. While several other experiences impact me, at the time of this reflection, these seem the most relevant and salient.

I currently work for an aging-in-place program, which informed much of my interest in this project. I work in a multigenerational team, with diverse backgrounds in multiple categories including race, education, sexuality, gender identity, languages spoken and comfortability with technology. The team formed mentorship bonds with one another, similar to those described in our focus groups and it has highlighted incredible amounts of resiliency and power building available to marginalized people when the larger communities they take part in share knowledge and resources.

**References for Supplementary Material**

Creswell, J. W., & Plano Clark, V. L. (2017). *Designing and Conducting Mixed Methods Research* (3rd ed.). Sage.

Flores-Flores, O., Otero-Oyague, D., Rey-Evangelista, L., Zevallos-Morales, A., Ramos-Bonilla, G., Carrión, I., Patiño, V., Pollard, S. L., Parodi, J. F., Hurst, J. R., Gallo, J. J., & Reynolds, R. (2023). Agency and mental health among Peruvian older adults during the COVID-19 lockdown. *The Journals of Gerontology, Series B*, *78*(6), 1109–1117. https://doi.org/10.1093/geronb/gbad040

Gorelick, S. (1991). Contradictions of feminist methodology. *Gender and Society, 5*(4), 459–477.

Lowers, J., Datcher, I., Kavalieratos, D., Hepburn, K., & Perkins, M. M. (2024). Proactive care-seeking strategies among adults aging solo with early dementia: A qualitative study. *The Journals of Gerontology, Series B*, *79*(5), 1–7. https:/doi.org/10.1093/geronb/gbae020

McCorkel, J.A & Myers K. (2003). What difference does difference make? Position and privilege in the field. *Qualitative Sociology, 26*(2), 199–231.

McHugh, M.C. (2014). Feminist qualitative research: Toward transformation of science and society. *The Oxford Handbook of Qualitative Research.*

Mertens, D. M. (2012). Transformative mixed methods: Addressing inequities. *American Behavioral Scientist*, *56*(6), 802–813. http://doi.org/10.1177/0002764211433797

Roller, M. R., & Lavrakas, P. J. (2015). *Applied Qualitative Research Design: A Total Quality Framework Approach*. The Guilford Press.
